# Growth Arrest-Specific 6 (Gas6) and TAM Receptors in Mouse Platelets

**DOI:** 10.4274/tjh.2013.0097

**Published:** 2015-02-15

**Authors:** Fikriye Uras, Burhanettin Küçük, Özlem Bingöl Özakpınar, Ahmet Muzaffer Demir

**Affiliations:** 1 Marmara University Faculty of Pharmacy, Department of Biochemistry, İstanbul, Turkey; 2 Trakya University Faculty of Medicine, Department of Hematology, Edirne, Turkey

**Keywords:** Blood platelets, Growth arrest-specific protein 6, Hemostasis

## Abstract

**Objective:**

Growth arrest-specific 6 (Gas6) is a newly discovered vitamin K-dependent protein, which is a ligand for TAM receptors [Tyro3 (Sky), Axl, and Mer] from the tyrosine kinase family. Gas6 knockout mice were resistant to venous and arterial thrombosis. There are contradictory reports on the presence of Gas6 and its receptors in mouse platelets. The objective of this study was to investigate whether Gas6 and its receptors were present in mouse platelets or not.

**Materials and Methods:**

Specific pathogen-free BALB/c male and female mice of 8-10 weeks old and 25-30 g in weight were anesthetized under light ether anesthesia and blood samples were taken from their hearts. RNAs were isolated from isolated platelets, and then mRNAs encoding Gas6 and TAM receptors were detected by reverse transcription-polymerase chain reaction (RT-PCR). Protein concentrations of Gas6 and TAM receptors in platelets were measured by ELISA, but not those of Mer, because of the absence of any commercial ELISA kit for mouse specimens.

**Results:**

RT-PCR results indicated the presence of mRNAs encoding Gas6 and Mer in mouse platelets. However, although RT-PCR reactions were performed at various temperatures and cycles, we could not detect the presence of mRNAs encoding Axl and Tyro3 (Sky). Receptor protein levels of Axl and Tyro3 were below the detection limits of the ELISA method.

**Conclusion:**

We found the presence of mRNAs encoding Gas6 and the receptor Mer in mouse platelets, but not Axl and Tyro3. Gas6, Axl, and Tyro3 protein levels were below the detection limits of the ELISA. The presence of mRNA is not obvious evidence of protein expression in platelets that have no nucleus or DNA. Further studies are required to clarify the presence of Gas6/TAM receptors in platelets using real-time PCR and more sensitive immunological methods, and future studies on mechanisms will indicate whether the Gas6/TAM pathway is a strategy for treatment of disorders.

## Abstract (tr)

**Amaç:**

Gas6 proteini (growth arrest specific 6), K vitaminine bağımlı protein ailesinin yeni bir üyesi olup reseptör tirozin kinazlardan TAM reseptörlerinin [Tyro3 (Sky), Axl ve Mer] bir ligandıdır. Gas6 geni susturulmuş farelerin, venöz tromboembolizme daha dirençli oldukları görülmüştür. Fare trombositlerinde Gas6 proteininin var olup olmadığı konusunda birbiriyle çelişkili yayınlar bulunmaktadır. Biz bu araştırmada, fare trombositlerinde Gas6 proteini ve reseptörlerinin varlığını hem mRNA hem de protein seviyesinde araştırmayı amaçladık.

**Gereç ve Yöntemler:**

Spesifik patojen içermeyen 8-10 haftalık, 25-30 gr ağırlığındaki Balb-C ırkı dişi/erkek fareler hafif eter anestezisi altında bayıltıp, kalpten kan örnekleri alındı. Önce trombositlerden mRNA’lar elde edildi. Daha sonra ters transkripsiyon-polimeraz zincir reaksiyonu (RT-PCR) yöntemiyle Gas6 ve TAM reseptörlerinin herbirini kodlayan mRNA’lar araştırıldı. Trombositlerdeki Gas6 ve TAM reseptör proteinleri ELISA yöntemiyle ölçüldü. Ancak farede Mer protein seviyesini ölçen bir ELISA kiti henüz üretilmediği için Mer seviyesi ölçülemedi.

**Bulgular:**

RT-PCR sonuçları fare trombositlerinde Gas6 ve Mer’i kodlayan mRNA’ların var olduğunu gösterdi. Çeşitli sıcaklık ve döngülerde RT-PCR deneyleri yapıldığı halde, Axl ve Tyro3’e (Sky) ait mRNA varlığı saptanamadı. Gas6, Axl ve Tyro3 (Sky) protein seviyeleri ELISA yöntemlerinin en düşük ölçüm sınırlarının altındaydı.

**Sonuç:**

Biz fare trombositlerinde, Gas6 ve onun reseptörlerinden sadece Mer’i kodlayan mRNA’ların var olduğunu bulduk ama Axl ve Tyro3’ü kodlayan mRNA’ların varlığını saptayamadık. Protein olarak Gas6, Axl ve Tyro3 seviyeri, ELISA yöntemlerinin ölçüm limitlerinin altındaydı. Trombositlerde mRNA’ların var olması, protein sentezinin burada yapıldığının kesin bir kanıtı değildir. Çünkü trombositlerde DNA yoktur. Trombositlerde Gas6/TAM reseptörlerinin varlığını daha iyi aydınlatmak için Gerçek Zamanlı-PCR ve yüksek duyarlılığa sahip immunolojik yöntemlerle yapılacak ileri araştırmalara ihtiyaç vardır. Bu moleküllerin fizyolojik mekanizması aydınlatıldığında hastalıkların tedavisinde yeni bir strateji olabilir.

## INTRODUCTION

The number of newly discovered vitamin K-dependent proteins has been increasing. These include osteocalcin, matrix Gla protein, Gla-rich protein, periostin, and growth arrest-specific gene 6 (Gas6). The Gas6 protein was first described as a vitamin K-dependent protein by Manfioletti et al. in 1993 [1]. Human Gas6 was mapped to chromosome 13q34 [2]. Gas6, a 75-kDa protein, has 44% sequence homology to natural anticoagulant protein S, but has no anticoagulant activity [1,3]. Both Gas6 and protein S have affinity to TAM receptors [Tyro3 (Sky), Axl, and Mer] from the receptor tyrosine kinase family [4]. Unlike the other vitamin K-dependent proteins, the primary site of synthesis for Gas6 is not the liver. However, it is expressed in endothelial cells [1], vascular smooth muscle cells [5], bone marrow cells [6], and normal and malignant hematopoietic cells [7].

Gas6 has structural domains: a carboxy-terminal domain that is similar to the sex hormone-binding globulin, 4 epidermal growth factor-like domains, and a γ-carboxylated amino-terminal domain (Gla domain) consisting of 11 glutamic acid residues [8,9]. The Gla domain, which forms complexes with calcium ions, is unique to vitamin K-dependent proteins. In the endoplasmic reticulum, some glutamate residues are posttranslationally modified by a vitamin K-dependent reaction [10]. Without the Gla domain the clotting factors cannot bind to phospholipid receptors on the surface of platelets. This binding is essential for the activation of the coagulation pathway [11,12,13]. In the absence of carboxylation, the coagulation process becomes defective [14,15]. In a conformationally specific manner, Gla residues can coordinate themselves to bind to cell membranes [16]. This interaction was shown to mediate both Gas6 and protein S binding to apoptotic cells [10].

It has been shown that Gas6 has a role in some physiological processes including cell growth arrest, bone resorption, phagocytosis of apoptotic cells, cell survival, cell proliferation, cell migration, and cell adhesion [1,17,18,19,20,21,22]. Gas6 binds to TAM receptors with markedly different affinities [23,24]. Axl was first isolated from chronic myelogenous leukemia patients in 1991 [25]. It was detected in some organs and cell lines including hematopoietic, mesenchymal, and epithelial cells [26].

In 1994, Tyro3 (Sky) was reported as a novel receptor [27]. The genomic structure is very similar to human Tyro3 and it is expressed in embryonic cells [28]. Mer was first identified in 1994 and its mRNA is present in bone marrow and monocytes [29,30].

Gas6 knockout mice experiments showed that these mice were resistant to venous and arterial thrombosis [31]. It was also shown that TAM receptor knockout mice were resistant to thrombosis and degradation of platelet aggregation [32]. It was suggested that Gas6 increases the tendency to thrombosis by leading to platelet plaque stabilization. The role of Gas6 and its receptors on platelets is uncertain.

There is disagreement as to whether synthesis of Gas6 in mouse platelets plays a role in these alterations. Some research groups have reported contradictory results for the presence of Gas6 and its receptors in mouse platelets. The presence of Gas6 and its 3 receptors was shown in both human and mouse platelets [31]. Chen et al. found the presence of Gas6 in mice platelets and only Mer from the TAM receptors [33]. Gould et al. showed the presence of each of the 3 receptors of TAM in mouse platelets [34]. It is obvious that there are contradictory findings related to the presence of Gas6 and its receptors in platelets. The objective of the present study was to clarify disagreement on the existence of Gas6 and TAM receptors in platelets.

## MATERIALS AND METHODS

### Mice

Specific pathogen-free BALB/c male and female mice of 8-10 weeks old and 25-30 g in weight were obtained from the Experimental Research and Animal Laboratory of Marmara University (İstanbul, Turkey). All of the experimental procedures were conducted according to the guidelines of the Animal Care and Use Committee of Marmara University.

### Isolation of Mice Platelets

Mice were anesthetized under light ether anesthesia and blood samples were collected from their hearts into tubes containing a 1:9 ratio of sodium citrate (3.2%). Platelet-rich plasma was isolated after centrifugation of whole blood at 200 x g for 10 min. The supernatant was transferred into an Eppendorf tube for centrifugation at 1000 x g for 10 min. Platelet pellets were suspended in a HEPES-Tyrode buffer (pH 7.4) containing 1 µg/mL prostacyclin and then centrifuged at 1000 x g for 10 min.

### Reverse Transcription-Polymerase Chain Reaction (RT-PCR)

Total RNA was isolated from platelets using a NucleoSpin RNA XS kit in line with the manufacturer’s instructions (Macherey-Nagel, Düren, Germany). The primers, obtained from Invitrogen, were as follows: those that generated a 400-bp Gas6 PCR product, 5′-CGG CAT TCC CTT CAA GGA GAG T-3′ (Gas6 forward; 1459-1480) and 5′-CTC AAC TGC CAG GAC CAC CAA CT-3′ (Gas6 backward; 1836-1868); those that generated a 238-bp Mer PCR product [35], 5′-GCA GGG ACT TAC AAA GAG CTT TCT-3′ (Mer forward; 1309-1332) and 5′-AGC CGA GGA TGA TGA ACA TAG AGT-3′ (Mer backward; 1542-1566); those that generated a 400-bp Axl PCR product [36], 5′-AGG CTC ATT GGC GTC TGT T-3′ (Axl forward; 2004-2033) and 5′-ATC GCT CTT GCT GGT GTA G-3′ (Axl backward; 2385-2403); and those that generated a 445-bp Tyro3 PCR product [37], 5′-GGA AGA GAC GCA AGG AGA C-3′ (Tyro3 forward; 1600-1620) and 5′-ATG GGA ATG GGG AGA CGA C-3′ (Tyro3 backward; 2027-2045). For each sample, 3 µL of total RNA was used. RT-PCR was performed with the QIAGEN OneStep PCR kit (QIAGEN, Düsseldorf, Germany) according to the manufacturer’s instructions. The same application for all genes was performed with reverse transcription at 50 °C for 30 min, PCR activation at 95 °C for 15 min, and final extension at 72 °C for 10 min. The following parameters for amplification were selected: Gas6, 38 cycles, denaturation at 94 °C for 30 s, annealing at 58 °C for 30 s, extension at 72 °C for 1 min; Mer, 35 cycles, same as the procedure for Gas6 except for annealing at 55 °C for 30 s; Tyro3, 38 cycles, same as the procedure for Gas6 except for annealing at 52 °C for 30 s; Axl, 35 cycles, same as the procedure for Gas6 except for annealing at 55 °C for 30 s. Using 2% agarose gel, PCR products were subjected to electrophoresis (100 V) and visualized with ethidium bromide (1%). These experiments were repeated 3 times with different sets of animals.

### Enzyme-Linked Immunosorbent Assay (ELISA)

Concentrations of Gas6, Axl, and Tyro3 in mouse plasma and platelets were measured with DuoSet ELISA kits (R&D Systems, Minneapolis, MN, USA) according to the manufacturer’s recommendations. Due to the lack of a commercial ELISA kit for Mer, we were unable to assay it. In brief, a 96-well microtiter plate was coated and incubated overnight at 4 °C with 100 µL of capture antibody. Phosphate-buffered saline Tween-20 (PBST) was used as the washing buffer, comprising phosphate-buffered saline (PBS) containing 0.1% Tween-20. All washing steps were carried out 5 times between steps. Wells were blocked with 1% bovine serum albumin (BSA) in PBST for 1.5 h at room temperature. Platelets were lysed by freezing/thawing [13] and 100 µL of calibrators or samples was added to the wells in 10-, 20-, or 40-fold dilutions. Wells were incubated for 1 h at 37 °C and the manufacturer’s instructions were then followed. The reaction was stopped after 20 min of incubation by addition of 100 µL of 2 N H2SO4. Optical density was measured at 450 nm on an ELISA reader.

### Statistical Analysis

Descriptive statistics were reported as median (25th percentile-75th percentile) for mouse plasma Gas6 concentrations.

## RESULTS

**Figure 1:**
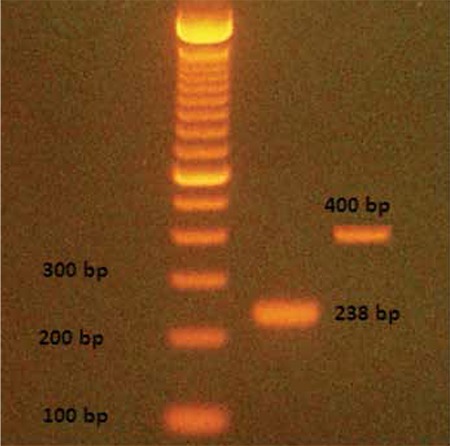
Expression of Gas6 (400 bp) and Mer (238 bp) gene amplifications in mouse platelet by reverse transcription-polymerase chain reaction (RT-PCR).

**Figure 2:**
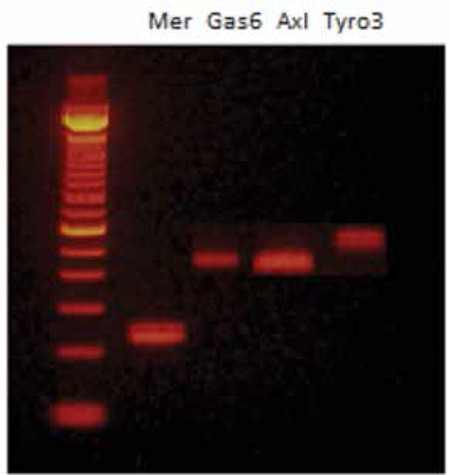
Expression of Gas6 (400 bp) and TAM receptors in mouse brain by reverse transcription-polymerase chain reaction (RT-PCR).

Platelets isolated in blood and flow cytometric analysis demonstrated the high purity (>93%) of the platelet preparation when we used CD41 as a marker. After isolation of total RNA from the platelets, the RT-PCR studies were carried out with suitable primers belonging to Gas6 and TAM receptors. Gas6 (400 bp) and Mer receptor (238 bp) gene amplification products were detected in mouse platelets, indicating the presence of mRNAs of Gas6 and Mer (Figure 1). However, for Axl and Tyro3, no appreciable PCR products were detected, which indicated no significant mRNA of Axl or Tyro3 in platelets. When we used brain tissue as a positive control, the primers and RT-PCR worked well for Axl and Tyro3 (Figure 2).

ELISA was used to measure protein levels of Gas6, Tyro3, and Axl in platelets. Mouse plasma was used for a positive control and PBS-BSA was used as a negative control. The analytical sensitivity of the assays was 0.7 ng/mL. Gas6 concentration was measured as 26 ng/mL (22.7-29.1) [median (25th percentile-75th percentile)] in the mouse plasma (n=6) and <0.7 ng/mL in platelets, which was lower than the detection limit and very close to the negative control value of PBS-BSA. These results showed us that the Gas6 protein level in the platelets was below the detection limit of ELISA. In the mouse platelets, the protein levels of Axl and Tyro3 were also below the detection limit (0.7 ng/mL) of the methods.

## DISCUSSION

Gas6 knockout mice studies showed that these mice were resistant to venous and arterial thrombosis and had normal hemostasis parameter values [31]. In parallel with these results, it was shown that TAM receptor knockout mice are resistant to thrombosis and degradation of platelet aggregation. It was suggested that Gas6 increases a tendency to thrombosis by leading to platelet plaque stabilization [32]. The role of Gas6 and its receptors on platelet functions is not clear.

The changes in platelet aggregation or activation have important roles in the pathophysiology of various diseases. There is disagreement as to whether synthesis of Gas6 in mouse platelets plays a role in these alterations. Some research groups have reported contradictory results for the presence of Gas6 and its receptors in mouse platelets. Angelillo-Scherer et al. showed the presence of Gas6 and its 3 receptors in both human and mouse platelets by RT-PCR [32]. In the current study, we found the presence of mRNAs encoding Gas6 and receptor Mer in mouse platelets, but not Axl or Tyro3. Protein Gas6, Axl, and Tyro3 levels were below the detection limits of the ELISA methods.

Chen et al. reported only the presence of receptor Mer in both human and mouse platelets using the western blot and RT-PCR methods [33]. They did not detect the presence of mRNAs for Axl and Tyro3. In Mer knockout mice, they observed impaired platelet functions and a decrease in the responsiveness to low-concentration agonists. Based on these findings, they considered that receptor Mer plays a role in the regulations of platelet functions. Sather et al. observed impaired platelet aggregation in Mer knockout mice [38].

Ishimoto and Nakano reported the presence of Gas6 in rat platelets by ELISA [39]. Moreover, in another study, Balogh et al. used mass spectroscopy and ELISA for measuring Gas6 in human plasma and platelets [40]. They reported that Gas6 levels were 13-23 ng/mL in human plasma by ELISA. However, in the same study they did not detect Gas6 protein in human platelets.

As a result of RT-PCR reactions, in the current study the presence of mRNAs of Gas6 and Mer was observed in platelets. It is known that platelets do not have DNA, but only RNA. Hence, the presence of mRNA is not obvious evidence of protein synthesis. Although RT-PCR reactions were performed at various temperatures and cycles, no visual band was detected for either Tyro3 or Axl. This agrees with the findings of Chen et al. [33].

One limitation of the current study is that we were unable to measure Mer levels as protein because of the absence of any commercial ELISA kit for mouse Mer. We found that the levels of Gas6 and receptors Axl and Tyro3 in platelets were below the measurable limits of ELISA.

Further studies are required to clarify the presence of Gas6 and TAM receptors in platelets using real-time PCR and more sensitive immunological methods, as well as studies of their effects on physiological mechanisms. The effects of Gas6 and TAM receptors in clot stabilization are a promising field of research. Further experiments in humans are required to form conclusions as to whether Gas6 may be a reliable pharmacological target in thrombosis, hemostasis, restenosis, and atherosclerosis.
